# Promotion of diet‐induced obesity and metabolic syndromes by BID is associated with gut microbiota

**DOI:** 10.1002/hep4.2052

**Published:** 2022-11-15

**Authors:** Shengmin Yan, Jun Zhou, Hao Zhang, Zhen Lin, Bilon Khambu, Gang Liu, Michelle Ma, Xiaoyun Chen, Naga Chalasani, Xiao‐Ming Yin

**Affiliations:** ^1^ Department of Pathology and Laboratory Medicine Tulane University School of Medicine New Orleans Louisiana USA; ^2^ Department of Pathology and Laboratory Medicine Indiana University School of Medicine Indianapolis Indiana USA; ^3^ Department of Emergency Medicine The Second Xiangya Hospital Central South University Changsha China; ^4^ Department of Medicine Indiana University School of Medicine Indianapolis Indiana USA; ^5^ Digestive Health Institute University of Illinois Urbana in Illinois USA

## Abstract

A growing body of evidence has indicated an expanding functional network of B‐cell lymphoma 2 (BCL‐2) family proteins beyond regulation of cell death and survival. Here, we examined the role and mechanisms of BH3 interacting‐domain death agonist (BID), a pro‐death BCL‐2 family member, in the development of diet‐induced metabolic dysfunction. Mice deficient in *bid* (*bid* ^*−/−*^) were resistant to high‐fat diet (HFD)–induced obesity, hepatic steatosis, and dyslipidemia with an increased insulin sensitivity. Indirect calorimetry analysis indicated that *bid* deficiency increased metabolic rate and decreased respiratory exchange ratio, suggesting a larger contribution of lipids to overall energy expenditure. While expression of several genes related to lipid accumulation was only increased in wild‐type livers, metabolomics analysis revealed a consistent reduction in fatty acids but an increase in certain sugars and Krebs cycle intermediates in *bid* ^*−/−*^ livers. Gut microbiota (GM) analysis indicated that HFD induced gut dysbiosis with differential patterns in wild‐type and in *bid* ^*−/−*^ mice. Notably, abrogation of GM by antibiotics during HFD feeding eliminated the beneficial effects against obesity and hepatic steatosis conferred by the *bid* deficiency. *Conclusion*: These results indicate that the protective role of *bid*‐deficiency against diet‐induced metabolic dysfunction interacts with the function of GM.

## Graphical abstract

1. Mice deficient in *bid* were resistant to high‐fat diet‐induced obesity, hepatic steatosis and dyslipidemia with an increased insulin sensitivity. 2. The protective role of *bid*‐deficiency against diet‐induced metabolic dysfunction interacts with the function of gut microbiota. 3. BID could affect lipid metabolism as suggested by the findings from gene expression profiling, indirect calorimetry and metabolomics.

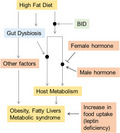

## INTRODUCTION

Obesity is associated with numerous diseases, including cardiovascular disease, nonalcoholic fatty liver disease (NAFLD), type 2 diabetes, and certain types of cancer.^[^ ^1^, ^2^ ^]^ Obesity‐associated conditions are the leading causes of premature death.^[^ ^3^ ^]^ In obesity‐associated conditions, NAFLD is a primary and common pathological presentation.^[^ ^4^ ^]^ Many factors can contribute to obesity and metabolic syndrome,^[^ ^5^ ^]^ including the BCL‐2 family proteins.^[^ ^6^ ^]^

B‐cell lymphoma 2 (BCL‐2) family proteins have canonical roles in cell death and survival, and functions beyond.^[^ ^6^, ^7^, ^8^ ^]^ For example, the pro‐death BH3‐only molecule, BH3 interacting‐domain death agonist (BID),^[^ ^9^, ^10^ ^]^ has also been involved in cell proliferation,^[^ ^11^, ^12^ ^]^ DNA damage response^[^ ^13^, ^14^ ^]^ and in innate immune responses.^[^ ^15^, ^16^ ^]^ Two other BH3‐only molecules, BCL‐2‐associated agonist of cell death (BAD)^[^ ^17^, ^18^, ^19^ ^]^ and BCL‐2‐interacting mediator of cell death (BIM),^[^ ^20^ ^]^ have also been found to participate in non‐apoptosis‐related function in metabolic regulation. How the BH3‐only BCL‐2 proteins regulate the various non‐apoptosis functions is not fully understood and could vary among individual members and the pathological context.^[^ ^18^, ^19^, ^20^, ^21^, ^22^ ^]^

Gut microbiota (GM) consists of a diverse community of symbiotic bacteria, which affect the health of the host in a number of ways, including through their metabolites.^[^ ^23^ ^]^ The critical role of GM in metabolic syndrome has been well studied in rodents and humans.^[^ ^24^, ^25^ ^]^ Indeed, the gut microbial metabolites can modulate metabolism in multiple tissues, thereby playing key roles in the pathogenesis of obesity, NAFLD, and type 2 diabetes.^[^ ^26^ ^]^ However, their interaction with the BCL‐2 family proteins was not known. Here we report that *bid*‐deficient mice are resistant to diet‐induced obesity and metabolic syndrome, which can be overcome by removing GM, thus revealing a pathway for the BCL‐2 family proteins to regulate metabolism.

## METHODS

### Animals and treatments

Mice deficient in *bid* (*bid* ^*−/−*^ mice) were created previously^[^ ^10^ ^]^ and had been backcrossed to C57BL/6 background for more than 15 generations. *Lep* ^*ob*^ (*Ob/Ob*) mice were purchased from the Jackson Laboratory and crossbred with *bid* ^*−/−*^ mice. C57BL/6 mice (wild‐type [WT]) were used as control mice. Male mice were maintained on a 12‐h dark/12‐h light cycle with free access to food and water. At 10 weeks of age, mice were provided *ad libitum* chow diet (RD), high‐fat diet (HFD; D12492, Research Diets, Inc.), or high‐fat and high‐carbohydrate diet (HFHCD; D12331, Research Diets, Inc.) for 10 to 24 weeks. For antibiotics treatment, mice (5–6 weeks old) were given antibiotics (ABX; 0.5 g/L neomycin sulfate and 1 g/L ampicillin) in drinking water for 5 weeks before HFD feeding. The water supply was renewed every 2 days and maintained during HFD feeding. For adeno‐associated virus (AAV)–mediated overexpression, *bid* ^*−/−*^ mice (10 weeks old) were given intravenously 2 × 10^11^ gas chromatography (GC)/mouse of AAV cluster of differentiation 36 (CD36) or AAV‐enhanced green fluorescent protein, followed by HFD feeding 1 week later. All animal experiments were approved by the Institutional Animal Care and Use Committee of Indiana University and Tulane University.

### Antibodies and chemicals

Antibodies and polymerase chain reaction (PCR) primers used in this study are listed in Tables S1 and S2, respectively.

### Metabolic cage study

WT and *bid* ^*−/−*^ mice were given HFD at 10 weeks old. After HFD feeding for 10 weeks, mice were placed in metabolic cages (LabMaster; TSE Systems, Inc.). Each cage was maintained at 25°C at a 12‐h dark/12‐h light cycle. After acclimatization individually for 72 h, the O_2_ consumption (VO_2_, ml/kg/min), CO_2_ production (VCO_2_, ml/kg/min), heat generation, physical activity, and food intake were measured every 10 min for a total of 48 h. Physical activity was measured by horizontal and vertical movement (XYZ‐axis). Average respiratory exchange ratio (RER) was calculated as the respiratory quotient (VCO_2_/VO_2_).

### Fecal 16S rRNA sequencing

Fecal samples were collected from mice before and after HFD feeding and stored at −80°C. Fecal DNA was extracted from frozen fecal samples using the E.Z.N.A. Stool DNA Kit (Omega Bio‐Tek, Inc.). All DNA samples were stored at −80°C before sequencing, which was performed by SeqMatic LLC using Illumina sequencing libraries. FASTQ data were processed on Illumina's BaseSpace servers using the Qiime pipeline. Relative abundance of all bacteria was calculated for further analysis. Principle coordinates analysis (PCoA) was conducted using multidimensional scaling function in SPSS for Windows 17.0 Software (SPSS, Inc.). Heatmaps were generated using Morpheus (https://software.broadinstitute.org/morpheus). Values in the heatmap were mapped to colors using the minimum and maximum of each row independently. The hierarchical cluster of each heatmap was performed using the one‐minus Pearson correlation method.

### Metabolomics analysis

Liver tissue (4 mg per sample) was used for analysis. The untargeted profiling of primary metabolism was analyzed by GC coupled with time‐of‐flight mass spectrometry (TOF‐MS) (West Coast Metabolomics Center, University of California at Davis). The sum of all peak heights for all identified metabolites for each sample was calculated, and raw data were normalized using the total average peak‐sums. Normalized data were analyzed using MetaboAnalyst 5.0 (https://www.metaboanalyst.ca/). Normalized data were also calculated as fold changes of RD‐fed WT group.

### Statistical analysis

The 16S sequencing data were represented as median with interquartile range. All other data were represented as means with standard errors (mean ± SEM). For 16S sequencing data, Mann–Whitney test was performed to identify bacteria with significantly different proportions between different groups. For all other data, to determine statistical significance, Student's *t* test was used to assess statistical differences between two groups. Statistical significance among multiple treatment groups was determined using one‐way analysis of variance followed by Duncan's *post‐hoc* test. Results were considered statistically significant for *p* value < 0.05. Statistical analyses were performed using SPSS for Windows 17.0 Software.

For all other methods see the Supporting Materials.

## RESULTS

### BID promotes diet‐induced obesity and metabolic syndrome

**FIGURE 1:**
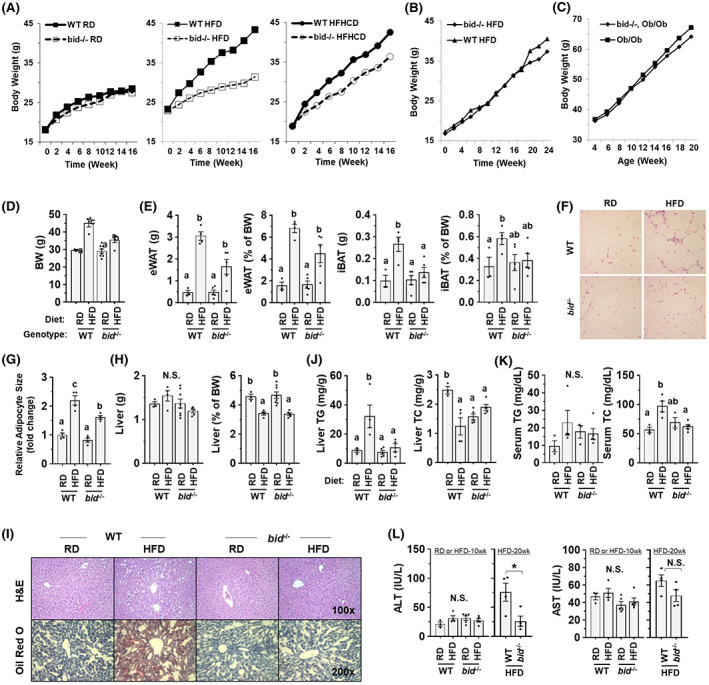
Mice that are deficient in BH3 interacting‐domain death agonist (*bid* ^*−/−*^) are resistant to high‐fat diet (HFD)–induced obesity, hepatic steatosis, dyslipidemia, and liver injury. (A) Male wild‐type (WT) and *bid*‐deficient (*bid* ^*−/−*^) mice were fed with regular chow diet (RD), HFD, or high‐fat high‐carbohydrate diet (HFHCD) for 16 weeks (n = 5–8). The body weight (BW) was measured every 2 weeks. (B) Female WT and *bid* ^*−/−*^ mice were fed with HFD for 24 weeks (n = 5–8). The body weight was measured every 2 weeks. (C) Male mice with the genotypes of *Ob/Ob* or *bid* ^*−/−*^:*Ob/Ob* were monitored for the body weight at different ages under RD (n = 5–8). (D) Male WT and *bid* ^*−/−*^ mice were fed with RD or HFD for 10 weeks. The body weight was measured at the end of 10 weeks. (E) Weight and the percentage of the body weight of epididymal white adipose tissue (eWAT) and interscapular brown adipose tissue (iBAT) in HFD‐fed WT and *bid* ^*−/−*^ mice. (F) Hematoxylin and eosin (H&E) staining of eWAT from mice with indicated genotypes and diet (×200). (G). Relative adipocyte size of eWAT with fold change over the WT‐RD group. (H) Liver weight and percentage of liver weight over body weight were measured in male mice with designated genotypes and given the indicated diets for 10 weeks. (I) Representative images of hepatic H&E staining (×100) and Oil Red O staining (×200). (J,K) Triglyceride (TG) and total cholesterol (TC) levels in the liver (C) and the serum (D) (n = 3–5). (L) Serum levels of alanine aminotransferase (ALT) and aspartate aminotransferase (AST) in male mice given the indicated diet for 10 or 20 weeks. Data are shown as means ± SEM. Data are shown as means ± SEM. Groups with different letters or asterisk had significant differences (*p* < 0.05). N.S., no statistical significance.

To investigate the role of BID in metabolism, we first measured the impact of *bid* deficiency on the body weight of mice given different types of diet. There was no significant difference between the WT and *bid* ^*−/−*^ mice in overall body weight gain under the RD regime (Figure 1A). However, *bid* ^*−/−*^ mice responded to HFD or HFHCD differently. Male *bid* ^*−/−*^ mice did not gain as much body weight as the WT mice in a 16‐week feeding regime (Figure 1A). Male *bid* ^*−/−*^ mice were resistant to diet‐induced obesity (DIO) as early as the start of HFD or HFHCD, with a significantly lower percentage gain of body weight in either food uptake regime. In contrast, female *bid* ^*−/−*^ mice acted much like the WT counterparts, with no resistance to body‐weight gain in the same 16‐week period, and only showed minor difference by week 24 (Figure 1B). It is notable that female mice did not gain as much weight as the male mice following HFD (Figure 1B vs. Figure 1A), reflecting a degree of resistance to DIO, compared with the male mice. Thus, it appears that *bid* deficiency could not offer the female mice additional resistance to DIO. We further crossed *bid* ^*−/−*^ mice to *Ob/Ob* mice, which developed the obesity due to lack of leptin and increased food uptake.^[^ ^27^ ^]^ Male mice with *bid* deficiency and *Ob/Ob* genotype gained the same body weight as the *Ob/Ob* mice (Figure 1C), both of which gained more than WT male mice fed with HFD, suggesting that *bid* deficiency–induced resistance to obesity was selective toward the effect of diet composition but not the amount of food consumed.

We then investigate the potential mechanism of this selective resistance to DIO using male mice undergoing a standard 10‐week HFD feeding regime (Figure 1D). We also confirmed that *bid* deficiency exhibited the resistance to the weight gain of both brown and white adipose tissues (Figure 1E). Histological examination clearly demonstrated an enlarged size of adipocytes in WT, but not in *bid* ^*−/−*^ mice (Figure 1F,G). Metabolic syndrome is a collection of symptoms, including obesity, NAFLD, dyslipidemia, and insulin resistance.^[^ ^24^ ^]^ We thus examined whether *bid* deficiency had an impact on the other presentation of metabolic syndrome. Although liver weight was not increased (Figure 1H), hepatic steatosis was notably in the WT but not in *bid* ^*−/−*^ mice, as measured by hematoxylin and eosin and Oil Red O staining (Figure 1I) and the level of hepatic triglyceride (TG) (Figure 1J). Total cholesterol (TC) level in the liver was not increased (Figure 1J). Correspondingly, dyslipidemia was notable in WT but not in *bid* ^*−/−*^ mice with elevated blood TG and TC (Figure 1K). Liver injury was not obvious as measured by the blood level of liver enzymes alanine aminotransferase and aspartate aminotransferase in the 10‐week HFD regime (Figure 1L). However, extension of HFD feeding to 20 weeks led to elevation of these enzymes in the blood (Figure 1L) in addition to hepatic steatosis and dyslipidemia (Figure S1A–C) in WT mice, but not in *bid* ^*−/−*^ mice, indicating the ability of the latter to resist the development of steatohepatitis and liver injury.

**FIGURE 2:**
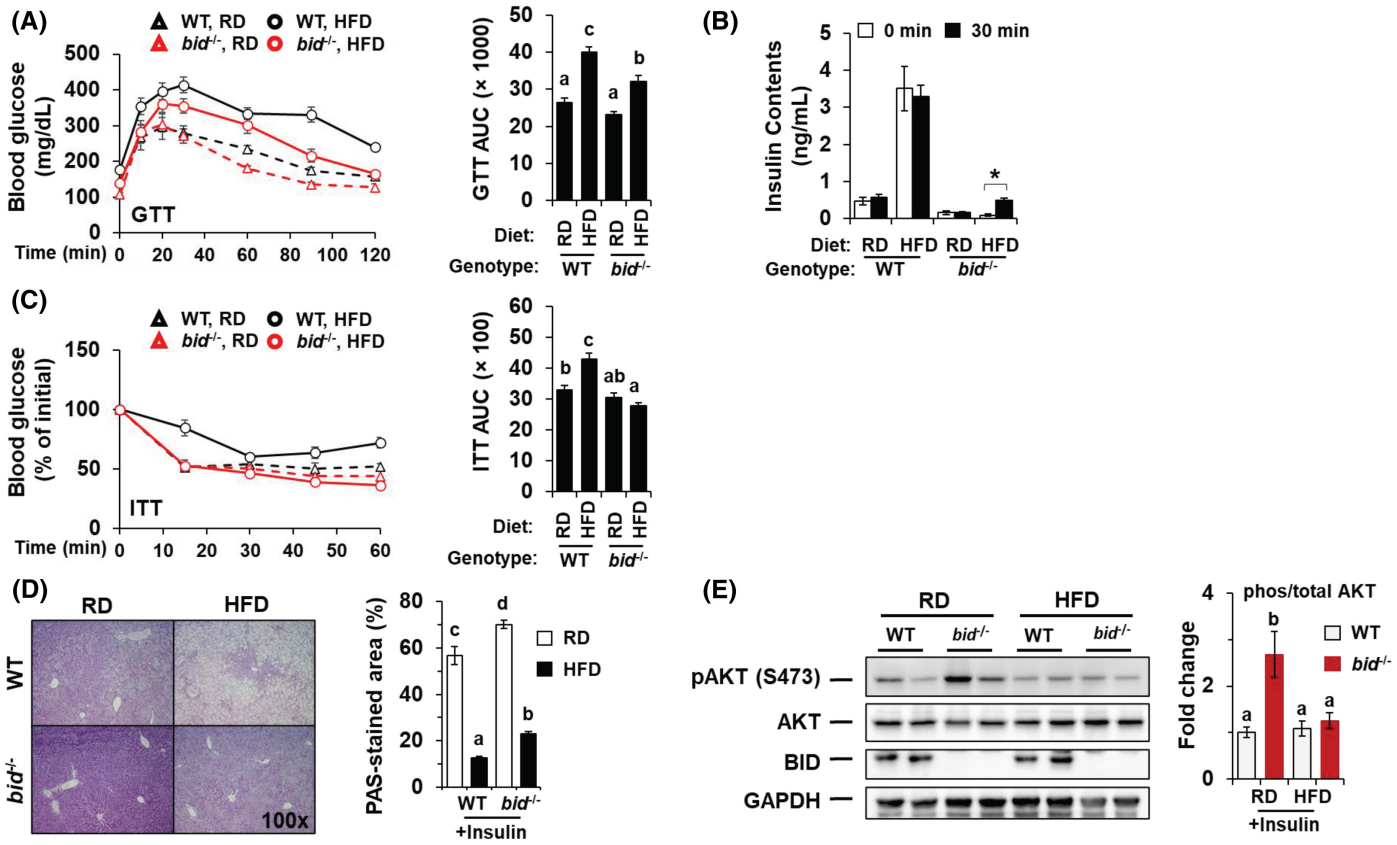
*bid* ^*−/−*^ mice demonstrate higher sensitivity to insulin than WT mice. Male WT and *bid*‐deficient (*bid* ^*−/−*^) mice on RD or HFD for 10 weeks were subjected to the following assays: glucose tolerance test (GTT) (A), measurement of serum levels of insulin before and after glucose challenge (B), insulin tolerance test (ITT) (C), measurement of hepatic glycogen levels with periodic acid–Schiff (PAS) staining (D), and phosphorylation of hepatic protein kinase B (AKT) (E) following insulin challenge. For (A) and (C), n = 4–5. For (D), percentage of positive area was quantified with Image‐J (n = 3). For (E), phosphorylation levels of AKT were calculated as the ratio of phosphorylated and total protein levels (phos/total) and expressed as fold change of WT mice with RD group (n = 3–4). Data were shown as means ± SEM. Groups with different letters or asterisks (B) had significant differences (*p* < 0.05). AUC, area under curve; GAPDH, glyceraldehyde 3‐phosphate dehydrogenase.

A major presentation of metabolic syndrome is insulin resistance, which is associated with the development of type 2 diabetes. Glucose tolerance test indicated that *bid* ^*−/−*^ mice had a faster rate of glucose clearance following HFD feeding (Figure 2A). Serum levels of insulin were significantly higher in HFD‐fed WT mice than in *bid* ^*−/−*^ mice (Figure 2B), suggesting a milder insulin resistance in the latter, which was confirmed by the insulin tolerance test (ITT). ITT showed a faster blood glucose clearance in HFD‐fed *bid* ^*−/−*^ mice than that in the HFD‐fed WT mice following insulin injection (Figure 2C).

While reducing blood glucose level by enhancing cellular uptake by peripheral tissues, insulin also enhances glycogen storage in hepatocytes.^[^ ^28^ ^]^ Following insulin administration, *bid* ^*−/−*^ livers stored more glycogen than WT livers (Figure 2D). This impact of *bid* deficiency was more notable in RD‐fed mice than in the HFD‐fed mice, where glycogen level was reduced due to suppressed synthesis via insulin resistance.^[^ ^28^, ^29^ ^]^ However, *bid* ^*−/−*^ livers retained more glycogen (Figure 2D), implying a better preservation of insulin sensitivity even in the HFD condition. One way that insulin stimulates glycogen synthase is through protein kinase B (AKT)–mediated suppression of glycogen synthase kinase 3 (GSK3), thus releasing glycogen synthase from the inhibition by GSK3.^[^ ^28^, ^29^ ^]^ Insulin elevates the level of phosphorylated AKT, an active form of AKT, which was significantly higher in *bid* ^*−/−*^ livers than that in WT livers under the RD (Figure 2E), consistent with the finding of differential glycogen levels in these mice (Figure 2D). However, the level of phosphorylated AKT was similar in WT and in *bid* ^*−/−*^ livers following HFD feeding (Figure 2E), suggesting that the impact of *bid* deficiency on glycogen synthesis under the HFD regime could be mediated by additional mechanisms other than the AKT signaling.

### BID deficiency altered HFD‐induced gut dysbiosis

To determine the contributing mechanisms by which BID affects diet‐induced metabolic syndrome, we investigated the role of gut dysbiosis, which has been widely considered as a critical factor for the development of metabolic syndrome.^[^ ^24^, ^30^, ^31^ ^]^ We examined fecal GM by 16S sequencing in mice before and after HFD feeding (Figure S2A). Before HFD feeding, the species diversity (Figure S2B) and number of identified species (Figure S2C) were similar in WT and *bid* ^*−/−*^ mice. PCoA analysis at species level also displayed a similar distribution for WT and *bid* ^*−/−*^ mice (Figure S2D), suggesting that deficiency of BID did not cause fundamental changes in GM. Dominant bacteria at the phylum level were Bacteroidetes, Firmicutes, and Proteobacteria in both WT and *bid* ^*−/−*^ mice before HFD feeding (Figure S2E), although the proportion of Actinobacteria and Verrucomicrobia was decreased in *bid* ^*−/−*^ mice (Figure S2F).

**FIGURE 3:**
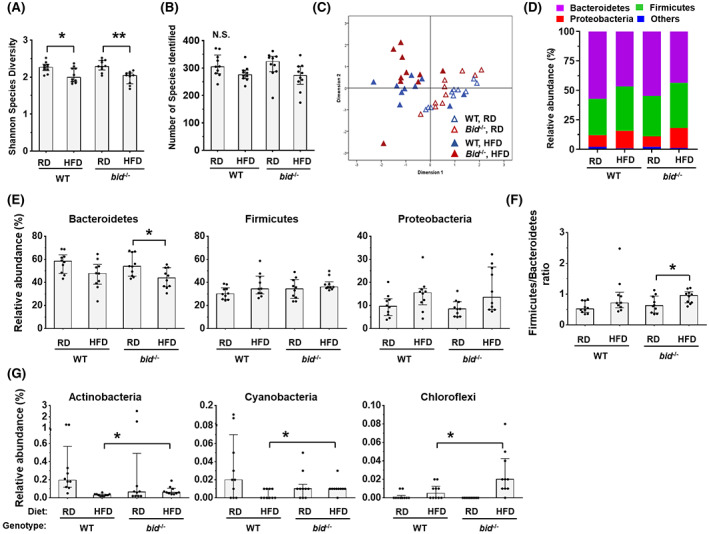
Alteration of gut microbiota (GM) in *bid* ^*−/−*^ mice after HFD feeding at the phylum level. Male WT and *bid*‐deficient (*bid* ^*−/−*^) mice were on RD or HFD for 10 weeks. (A,B) Shannon species diversity (A) and the number of identified S16 species (B) were determined in post‐experiment fecal samples. (C) Principal coordinates analysis (PCoA) based on relative abundance at species level shows a distinguishable profile of GM between HFD and RD mice. (D,E) Proportion of dominant bacteria (D) and relative abundance of major bacteria (E) at the phylum level in post‐experiment fecal samples. (F) Ratio of Firmicutes and Bacteroidetes in post‐experiment fecal samples. (G) Proportion of Actinobacteria, Cyanobacteria, and Chloroflexi was higher in HFD‐fed *bid* ^*−/−*^ mice compared with HFD‐fed WT mice. Data are shown as median with interquartile range, n = 10/group. Mann–Whitney test; *p < 0.05.

Following HFD feeding, fecal GM displayed a decreased species diversity (Figure 3A) and a lower number of identified species in both WT and *bid* ^*−/−*^ mice (Figure 3B). PCoA analysis suggested that data from different fecal samples were notably separated by diet but not genotype (Figure 3C). At the phylum level, HFD significantly decreased the proportion of Bacteroidetes and noticeably increased the proportion of Proteobacteria in mice (Figure 3D,E). The Firmicutes/Bacteroidetes ratio was thus increased following HFD feeding as reported previously,^[^ ^32^ ^]^ but the change was more significant in *bid* ^*−/−*^ mice (Figure 3F). In addition, we found a higher proportion of Actinobacteria, Cyanobacteria, and Chloroflexi in HFD‐fed *bid* ^*−/−*^ mice compared with HFD‐fed WT mice (Figure 3G), suggesting that deficiency of *bid* can cause differential alterations of gut bacteria in response to HFD at the phylum level.

We then interrogated the 16S sequencing results in more detail at the level of genus and species levels. There was no dramatic disproportion of GM in RD‐fed mice, although 11 low‐abundance bacteria at the genus level (Figure S3A) and 17 low‐abundance bacteria at the species level (Figure S3B) had already been altered in *bid* ^*−/−*^ mice, compared with those in WT mice. Following HFD feeding, 78 bacteria were disproportionated at the genus level (Figure S4A), in which 19 bacteria were differentially altered between WT and *bid* ^*−/−*^ mice (Figure S4B). Notably, two bacteria, *Lactobacillus* and *Rhodothermus*, were high‐abundance genus (Figure S4C), while the other 17 bacteria were in low‐abundance (Figure S4D). Similar to the finding at the genus level, 149 bacteria were altered at the species level in HFD‐fed mice (Figure S5A), in which 31 of them showed different patterns between WT and *bid* ^*−/−*^ mice (Figure S5B). Interestingly, six bacteria were oppositely altered by HFD in WT versus *bid* ^*−/−*^ mice (Figure S5C). It is worth noting that two high‐abundance species, *Bacteroides chinchillae* and *Bacteroides sartorii*, were enriched in HFD‐fed WT mice but not in HFD‐fed *bid* ^*−/−*^ mice.

Taken together, these results suggest that *bid* deficiency leads to a differential alteration of bacteria at the phylum, genus and species levels, particularly after HFD feeding.

### GM participates in the resistance of BID deficiency to diet‐induced obesity and hepatic steatosis

**FIGURE 4:**
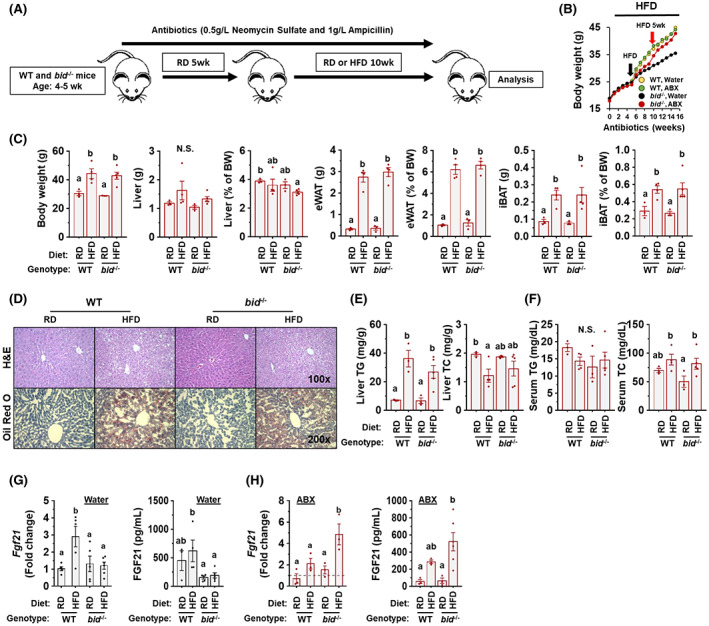
Resistance of *bid* ^*−/−*^ mice to diet‐induced obesity (DIO) and hepatic steatosis is eliminated with antibiotics (ABX) treatment. (A) Scheme of HFD feeding in combination with ABX treatment. (B) Changes of body weight during HFD feeding (black arrow indicates the beginning of HFD feeding; red arrow indicates 5 weeks after HFD feeding). (C) The weight of the whole body (BW), the liver, the eWAT, and the iBAT in HFD‐fed WT and *bid* ^*−/−*^ mice under ABX. (D) Representative images of hepatic H&E staining (×100) and Oil Red O staining (×200). (E,F) Triglyceride (TG) and total cholesterol (TC) levels in the liver (E) and the serum (F). (G) Hepatic messenger RNA (mRNA) level of fibroblast growth factor 21 (*fgf21*) and serum level of FGF21 in mice with designed genotype and diet. (H) Hepatic mRNA level of *fgf21*, and serum level of FGF21 in mice with designed genotype and diet in combination with ABX treatment. Data are shown as means ± SEM. Groups with different letters had significant differences (*p* < 0.05); one‐way analysis of variance (ANOVA) followed by Duncan's *post‐hoc* test.

The differential gut dysbiosis could still contribute to the differential metabolic patterns seen in *bid* ^*−/−*^ mice. We therefore treated mice with ABX before and during HFD feeding (Figure 4A). ABX treatment did not alter the pattern of body‐weight change in HFD‐fed WT mice but reversed the resistance of *bid* deficiency to HFD‐induced obesity (Figure 4B). Thus, the weight of the body and that of the adipose tissues were increased comparably in HFD‐fed WT and *bid* ^*−/−*^ mice without significant difference (Figure 4C). A similar level of liver weight (Figure 4C) and hepatic steatosis (Figure 4D,E) were also noted between HFD‐fed WT and *bid* ^*−/−*^ mice following ABX treatment. ABX treatment of HFD‐fed *bid* ^*−/−*^ mice also elevated serum cholesterol levels as in the WT mice (Figure 4F).

Because *bid* ^*−/−*^ mice given HFD showed resistance to obesity, which was abrogated by ABX, we housed HFD/ABX‐treated *bid* ^*−/−*^ mice with *bid* ^*−/−*^ mice that had not been exposed to HFD/ABX (Figure S6A). This experiment could allow the ABX‐treated *bid* ^*−/−*^ mice to pick up GM from the non‐HFD/ABX‐treated *bid* ^*−/−*^ mice via the feces excreted by the latter. The results showed that co‐housing reduced the elevation of the weight of the body, the adipose tissue, and the liver in HFD/ABX‐treated mice (Figure S6B–E). In addition, hepatic triglyceride level and serum cholesterol level (Figure S6F–I) were also reduced in these mice. These changes were similar to those in HFD‐fed *bid* ^*−/−*^ mice with no ABX treatment (Figure 1), suggesting that GM from *bid* ^*−/−*^ mice can reverse the impact of ABX.

Fibroblast growth factor 21 (FGF21) is expressed primarily by the liver and can be induced by stress and pathological process, such as steatosis.^[^ ^33^ ^]^ In humans, serum concentration of FGF21 is increased in diabetes, obesity, NAFLD, and metabolic syndrome.^[^ ^34^ ^]^ Hepatic *Fgf21* expression and serum FGF21 levels (Figure 4G) were increased in WT mice following HFD feeding as expected, but not in the same treated *bid* ^*−/−*^ mice. The increase of FGF21 in HFD‐fed WT mice could be a response to reduced FGF21 sensitivity.^[^ ^34^ ^]^ Because *bid* deficiency conferred a relatively normal metabolic condition following HFD feeding, lower levels of FGF21 could be another manifestation of the resistance of *bid* ^*−/−*^ mice to diet‐induced hepatic steatosis. Consistently, ABX was able to elevate the hepatic expression of *Fgf21* and serum level of FGF21 in HFD‐fed *bid* ^*−/−*^ mice (Figure 4H), due to the elimination of the benefits of *bid* deficiency, and the development of fatty liver condition in these mice.

Overall, these results demonstrate that gut dysbiosis contributes to the resistance of *bid* ^*−/−*^ mice to HFD‐induced obesity and hepatic steatosis, which was reversed by ABX‐treatment. Differences in the expression of FGF21 following HFD feeding may contribute to the different metabolic phenotypes in the WT and *bid* ^*−/−*^ mice.

### BID deficiency led to an altered lipid metabolism

**FIGURE 5:**
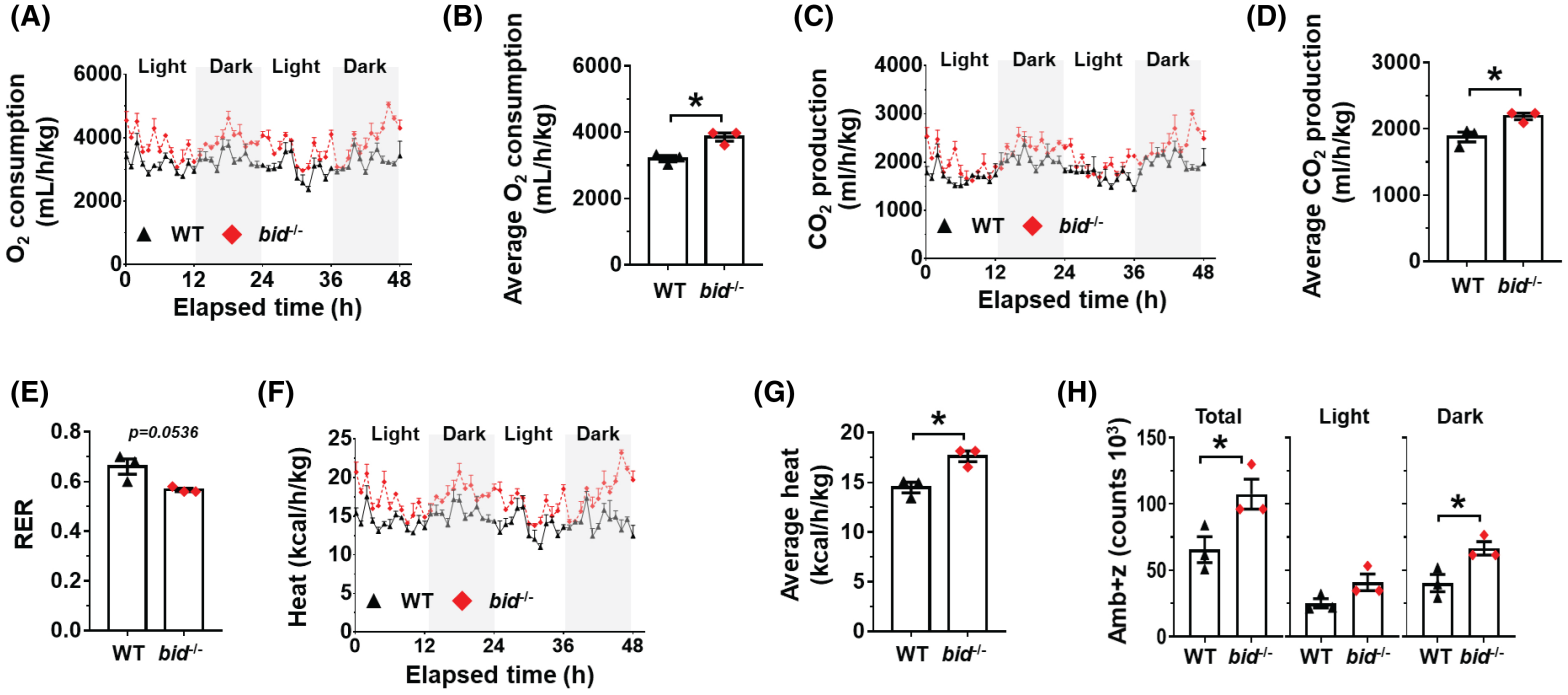
HFD‐fed *bid* ^*−/−*^ mice have a metabolic pattern favoring low body mass. Male WT and *bid*‐deficient (*bid* ^*−/−*^) mice (n = 3) were fed with HFD for 10 weeks and subjected to indirect calorimetry analysis for 48 h after initial acclimation. (A–D) Dynamic O_2_ consumption (A), average O_2_ consumption (B), dynamic CO_2_ generation (C), and average CO_2_ generation (D) were measured. (E) The respiratory exchange ratio (RER) was calculated by the ratio between the average of CO_2_ generation and the average consumption of O_2_. (F,H) Dynamic heat generation (F), average heat generation (G), and average physical activity (H) were recorded. Physical activity was represented by the ambulatory activity in three axes (Amb+Z) during a 48‐h period. Light time period was from 7:00 to 19:00; dark time period was from 19:00 to 7:00. Data are shown as means ± SEM. Groups with asterisks had significant differences (*p* < 0.05).

To investigate the metabolic phenotypes of mice, we subjected mice fed with HFD for 10 weeks to indirect calorimetry analysis. Both O_2_ consumption (Figure 5A,B) and CO_2_ production (Figure 5C,D) were significantly increased in *bid* ^*−/−*^ mice. The RER was lower in HFD‐fed *bid* ^*−/−*^ mice (Figure 5E), suggesting a metabolism favoring fatty acids as the more dominant contributor of the overall energy expenditure.^[^ ^35^, ^36^ ^]^ Moreover, both heat generation (Figure 5F,G) and activity (Figure 5H) were increased in HFD‐fed *bid* ^*−/−*^ mice, which may promote energy use. The calorimetric results indicate that *bid* deficiency alters energy metabolism in HFD‐fed mice, which favors reduction of body fat composition.

**FIGURE 6:**
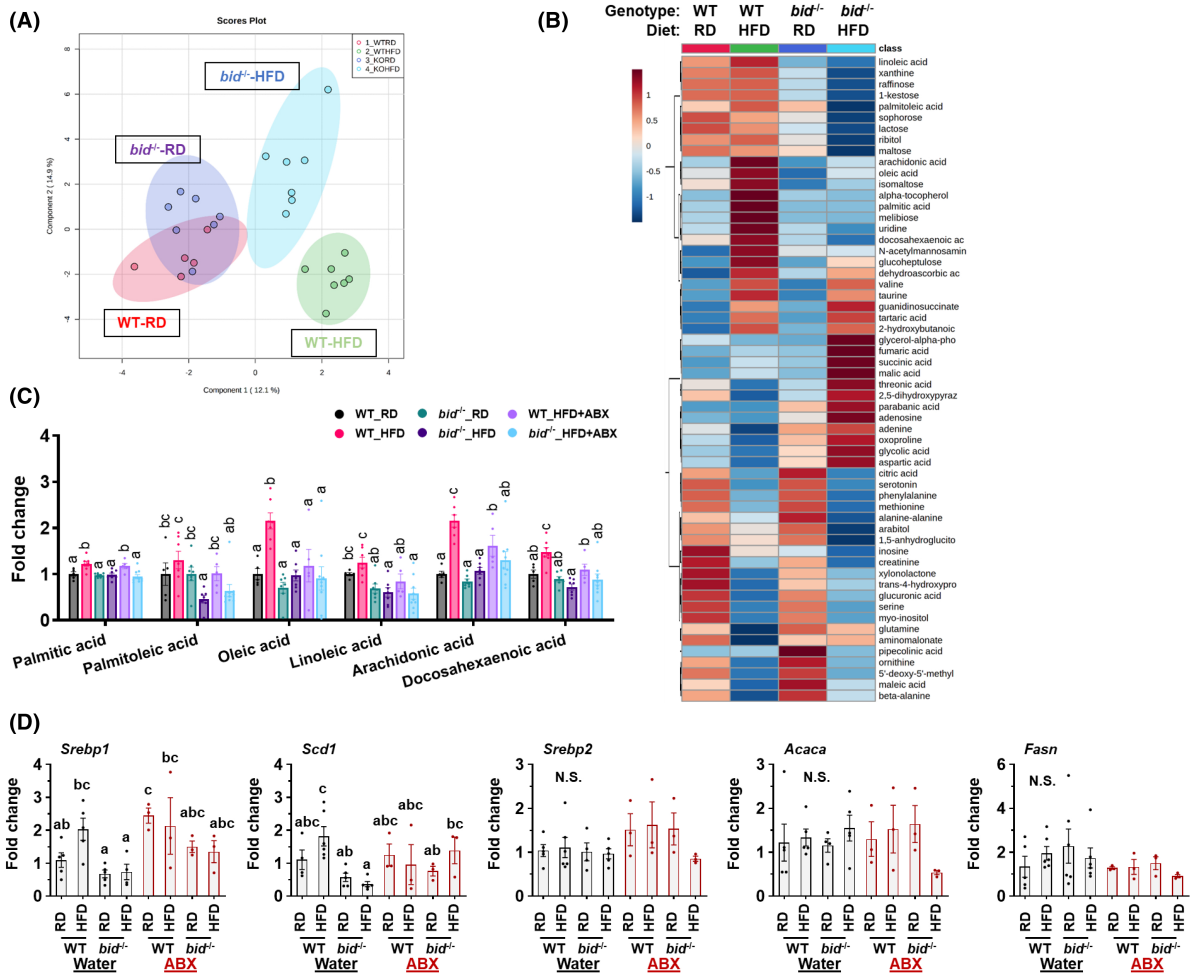
HFD‐fed *bid* ^*−/−*^ mice have altered hepatic metabolite profiles in lipid metabolism. Male WT and *bid* ^*−/−*^ mice were on RD or HFD for 10 weeks with or without ABX treatment (Figure 4A). (A) Livers from RD‐fed or HFD‐fed mice were subjected to metabolomics. Identified metabolites were subjected to sparse partial least‐squares discriminant analysis (sPLS‐DA). (B) Heat map shows metabolites with significant level changes among four groups (ANOVA; *p* < 0.05). sPLS‐DA plots and heat map were generated by MetaboAnalyst 5.0. Data are shown as average log2 bin. (C) The levels of certain fatty acids in WT or *bid* ^*−/−*^ livers following RD or HFD in mice given water or ABX treatment. (D) Hepatic expression of genes related to lipid synthesis was assessed with or without ABX treatment. Data are shown as means ± SEM. Statistical analysis was performed by one‐way ANOVA followed by Duncan's *post‐hoc* test. Groups with different letters had significant differences (*p* < 0.05). *Acaca*, acetyl‐CoA carboxylase alpha; *Fasn*, fatty acid synthase; *Scd1*, stearoyl‐CoA desaturase 1; *Srebp1/2*, sterol regulatory element‐binding transcription factor 1/2.

To determine the impact of BID on hepatic metabolism on a broad basis, we performed untargeted profiling of primary metabolism using GC/TOF‐MS. The metabolites profiles in WT and *bid* ^*−/−*^ livers were largely overlapped under the RD condition but were clearly separated following HFD feeding (Figure 6A). Among the 152 identified metabolites (Table S3), the levels of 58 metabolites were significantly altered by the genotypes and/or the diet (Figure 6B). In particular, we noted that the level of several fatty acids, including palmitic acids, oleic acids and linoleic acids, was increased in WT livers, but not in *bid* ^*−/−*^ livers, following HFD (Figure 6C), consistent with the elevated fatty acid synthesis and the more severe fatty liver phenotype in the WT mice. Intriguingly, ABX treatment did not appear to significantly reverse the impact of *bid* deficiency on the individual level of these detected fatty acids. The individual fatty acid levels remained relatively low in HFD‐fed *bid* ^*−/−*^ livers with or without ABX treatment, implying that these changes were not solely the target of the GM in leading to steatosis.

To further determine the impact of *bid* deficiency on lipid metabolism, we examined the hepatic expression of genes related to lipid metabolism. HFD induced the expression of lipid synthesis genes, including sterol regulatory element‐binding transcription factor 1 (*Srebp1*) and stearoyl‐CoA desaturase 1 (*Scd1*), in WT but not in *bid* ^*−/−*^ livers (Figure 6D). The basal levels of these two genes under RD were also lower in *bid* ^*−/−*^ mice. The gene‐expression levels correlated with the level of fatty acids in the liver (Figure 6C) and the degree of steatosis (Figure 1). Moreover, the differences in the expression of *Srebp1* and *Scd1* between the WT and *bid* ^*−/−*^ livers was blunted by ABX treatment and became statistically insignificant (Figure 6D). These results suggest that the relatively lower capability of fatty acid synthesis in the *bid* ^*−/−*^ livers were corrected noticeably by ABX treatment.

**FIGURE 7:**
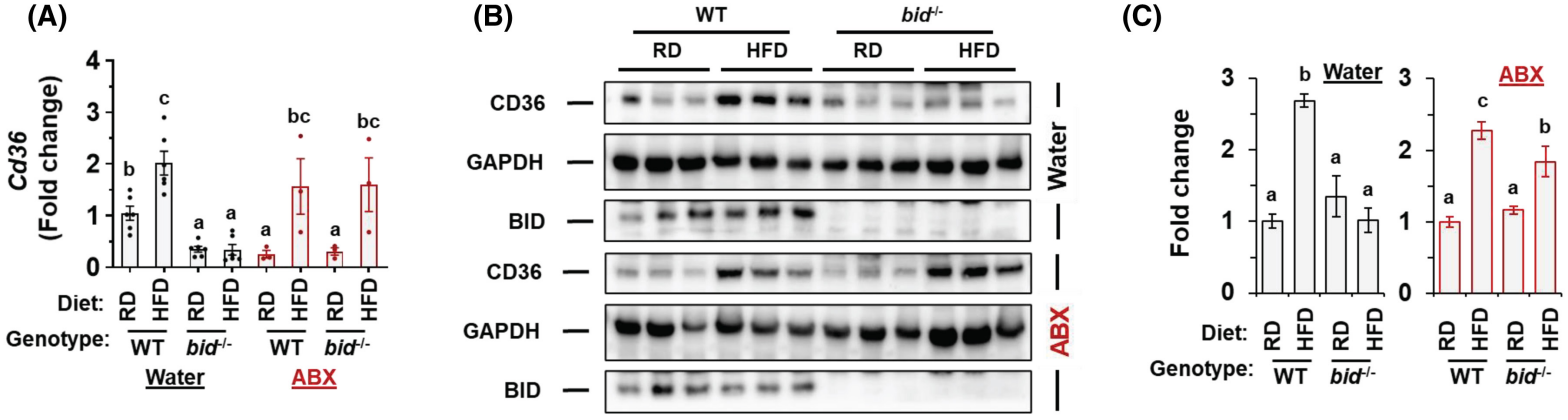
*bid* ^*−/−*^ mice were resistant to increase of hepatic CD36 induced by HFD. Male WT and *bid* ^*−/−*^ mice were on RD or HFD for 10 weeks with or without ABX treatment (Figure 4A). (A) Expression of cluster of differentiation 36 (*Cd36*) mRNA in the liver following HFD feeding. (B,C) Hepatic CD36 protein levels were analyzed by immunoblotting (B) and densitometry (C) in mice following HFD feeding in the absence or presence of ABX. Data are shown as means ± SEM. Statistical analysis was performed by one‐way ANOVA followed by Duncan's *post‐hoc* test. Groups with different letters had significant differences (*p* < 0.05).

No significant or consistent changes were observed in key genes related to lipid oxidation–related gene (Figure S7A), lipolysis (Figure S7B), and bile acid metabolism (Figure S7C), with or without ABX treatment. Although we had noted that there were some differences in the expression levels of lipoprotein lipase and fatty acid binding protein 1 between WT and *bid* ^*−/−*^ livers in relation to ABX treatment (Figure S7D), the most significant and consistent changes related to lipid transportation were observed for the expression of CD36. CD36 is a class B scavenger receptor that can transport fatty acids into cells, and its expression level is increased in steatotic livers in both rodents and human.^[^ ^37^ ^]^ A beneficial effect of CD36 deletion in overcoming metabolic dysfunction has been observed in both *Cd36*‐constitutive and liver‐specific *Cd36* knockout mice,^[^ ^38^, ^39^ ^]^ although deletion of CD36 in *Ob/Ob* mice exacerbated hepatic steatosis.^[^ ^40^ ^]^ We found that *Cd36* expression was low and was not elevated by HFD in *bid* ^*−/−*^ livers as in the WT mice (Figure 7A–C), implying that there were fewer fatty acids being transported to the hepatocytes. On the other hand, ABX caused the elevation of *Cd36* expression in *bid* ^*−/−*^ livers following HFD at both the messenger RNA (Figure 7A) and protein level (Figure 7B,C), which could suggest an increased fatty acid transport into the liver under ABX treatment. To explore the functional role of CD36, we exogenously overexpressed the *Cd36* gene alone in the *bid* ^*−/−*^ livers (Figure S8A–C). Although this overexpression led to noticeable but not statistically significant increase in the level of *Srebp1* and *Scd1* (Figure S8C), it did not appear to be able to overcome the resistance of the *bid* ^*−/−*^ mice to obesity (Figure S7D) or to hepatic steatosis (Figure S8E,F) or dyslipidemia (Figure S8G) following HFD feeding, suggesting that CD36 could be a contributing factor to, but not a sufficient cause for, the HFD‐resistant phenotypes of the *bid* ^*−/−*^ mice, which was also consistent with the context‐dependent effect of CD36 as shown previously.^[^ ^38^, ^39^, ^40^ ^]^

### BID deficiency led to an altered sugar and intermediate metabolism

**FIGURE 8:**
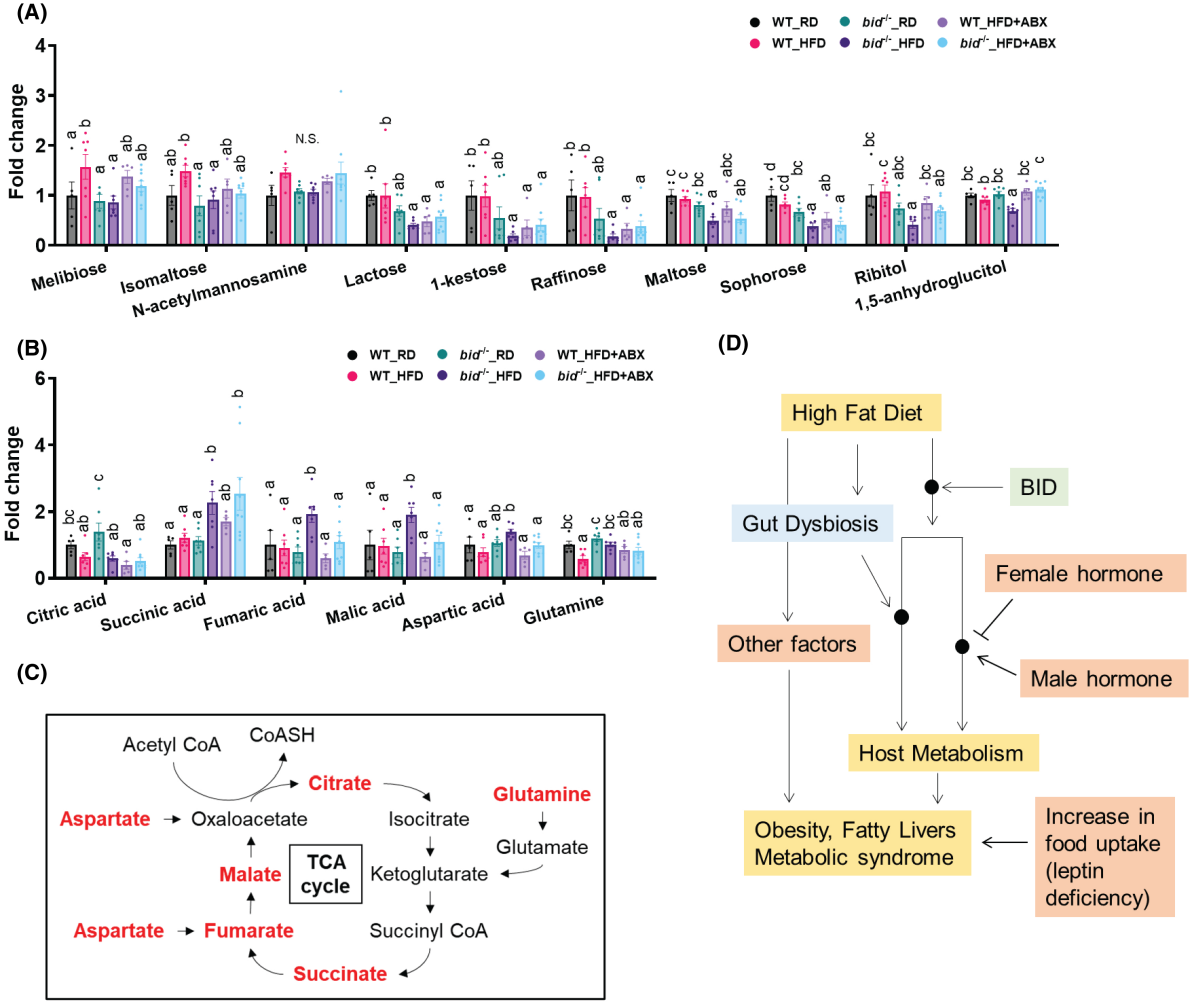
HFD‐fed *bid* ^*−/−*^ mice have altered hepatic profiles of intermediate metabolites. Male WT and *bid* ^*−/−*^ mice were on RD or HFD for 10 weeks with or without ABX treatment (Figure 4A). (A,B) The levels of certain sugar metabolites (A) or intermediate metabolites (B) in WT or *bid* ^*−/−*^ livers following RD or HFD, with or without ABX treatment. (C) A simplified diagram of trichloroacetic acid (TCA) cycle showing the conversion of the relevant intermediate metabolites. (D) A working model of the interplay among the various factors that can affect diet‐induced obesity and metabolic syndrome. Black dots indicate unknown mechanisms by which BID, altered microbial, and possibly other factors could affect the host under HFD. It is possible that BID could modify the host response to altered microbial, leading to changes in metabolism of lipids and other intermediate metabolites, which ultimately affect the obesity phenotype. The pathway regulated by sex hormones, but not that by increased food uptake (leptin deficiency), may likely converge with that of BID. Data are shown as means ± SEM. Statistical analysis was performed by one‐way ANOVA followed by Duncan's *post‐hoc* test. Groups with asterisks had significant differences (*p* < 0.05). CoA, coenzyme A.

Other than the changes in lipid metabolites, this metabolomics study also found that levels of several sugars were also elevated in the HFD‐fed WT mice, but not in *bid* ^*−/−*^ livers, such as melibiose, isomaltose and N‐acetylmannosamine (Figure 8A), whereas several other sugars were reduced in *bid* ^*−/−*^ livers, particularly following HFD, such as lactose, 1‐kestose, and raffinose. Interestingly, ABX treatment reverses the levels of many of these sugars, suggesting that GM has a large impact on their metabolism, which may indirectly affect the HFD‐induced hepatic presentation.

In contrast to the lower levels of fatty acids and sugars in the *bid* ^*−/−*^ livers, the level of several intermediate metabolites related to the Krebs cycle was higher in *bid* ^*−/−*^ livers than in the WT livers following HFD feeding (Figure 8B,C), suggesting a more robust metabolic activity in this pathway in the absence of BID. Notably, the elevated hepatic levels of fumarate, malate, aspartate, and glutamine were reversed to a different degree by ABX treatment in HFD‐fed *bid* ^*−/−*^ mice (Figure 8B), suggesting that the metabolism of these amino acids is more closely correlated with the metabolic pattern of the *bid* ^*−/−*^ mice. Taken together, changes in certain intermediate metabolites in the Krebs cycle can contribute to the metabolic syndrome in a way crossed with GM.

## DISCUSSION

BCL‐2 family proteins were initially discovered through their function in regulating apoptosis.^[^ ^7^ ^]^ BID belongs to the BH3‐only subfamily of the BCL‐2 molecules and is known to possess a pro‐apoptosis and pro‐proliferation function.^[^ ^9^, ^10^, ^11^ ^]^ We show here that BID has a function in promoting diet‐induced obesity and metabolic syndrome. Nonapoptotic functions of BCL‐2 family proteins have been reported for several members.^[^ ^7^, ^8^, ^17^, ^18^, ^20^, ^22^ ^]^ However, our studies indicate that the metabolic regulation function of BID is unique in its interaction with GM.

HFD feeding can cause gut dysbiosis,^[^ ^24^, ^30^, ^31^ ^]^ which is seen in both WT and *bid* ^*−/−*^ mice without significant differences, except certain microbes appeared to be differentially altered by HFD in WT and *bid* ^*−/−*^ mice. This evidence suggests that BID‐mediated metabolic changes may produce metabolites that may modulate GM in HFD‐fed mice. The connections among GM, the liver, and metabolic dysfunction have been documented in both mouse models and obese patients.^[^ ^23^, ^24^, ^25^, ^26^, ^32^ ^]^ In the present study, BID appears to play a detrimental role, as deletion of this *bid* gene renders the mice resistant to DIO. Interestingly, GM abrogation by antibiotics eliminates the beneficial effect of *bid* deficiency, which is partially reversed by co‐housing ABX‐treated *bid*‐deficient mice with non‐ABX‐treated *bid*‐deficient mice, indicating that the functional role of BID in metabolic homeostasis interacts with the function of GM. Multiple mechanisms (Figure 8D) could be involved in the BID‐affected DIO and metabolic syndrome (Figures 1 and 2), a large part of which is dependent on GM (Figure 4), which is altered by HFD (Figure 3). There are diet‐induced changes that are affected by BID but are independent of GM, such as the level of certain metabolites (Figures 6A and 8B). Interestingly, female *bid* ^*−/−*^ mice were not more resistant to DIO than female WT mice (Figure 1B). Previous studies show that female mice are protected against HFD‐induced metabolic syndrome and inflammatory response, which is likely related to female sex hormones.^[^ ^41^ ^]^ Our results were consistent with this observation that female mice do not gain as much weight as the male mice following HFD (Figure 1B vs. Figure 1A). Thus, it appears while *bid* deficiency can offer male mice some resistance to DIO, it could not offer the female mice additional resistance to DIO, suggesting that the DIO‐regulating pathway mediated by female hormones may converge at some point with that of BID, and that pathway, as discussed previously, could be still related to metabolism of lipids and intermediate metabolites (Figure 8D). Finally, BID does not regulate obesity induced by increased food update as manifested by the effect of leptin (Figure 1C), suggesting that its effect toward the composition of the food, high fat, can be overcome by the amount of food.

It appears that BID could affect the metabolic pattern following HFD feeding so that *bid* deficiency favors the use of lipids as the source of energy expenditure, as suggested by a lower RER.^[^ ^35^, ^36^ ^]^ Consistently, hepatic expression of multiple genes related to lipid metabolism, such as *Srebp1*, *Scd1*, *Fgf21*, and *Cd36*, was elevated in HFD‐fed WT mice but not in *bid* ^*−/−*^ mice. Notably, ABX treatment abrogated the difference between the WT and *bid* ^*−/−*^ mice in the expression of these genes (Figure 6E) and hepatic steatosis, suggesting a mechanistic connection among BID, GM, and lipid metabolism under the overfed condition. It seems plausible that GM may provide beneficial effects, which are antagonized by the effect of BID so that deletion of *bid* allows the manifestation of the beneficial effects, which are removed by ABX. How the two pathways of BID and GM interact is not known. Other than the intestine, the liver could be another important organ in these interactions. The gut–liver axis is well documented for the mutual interactions and effects between the two organs.^[^ ^42^ ^]^ Bile acids from the liver is important to lipid metabolism and depends on GM for processing. Both the level of bile acids and the type of diet shape the gut flora, which in turn affect the metabolism. The metabolites generated by the GM, including some short chain fatty acids, can affect hepatic metabolisms, particularly in the presence of HFD.^[^ ^43^ ^]^ Indeed, metabolomics studies indicate that the level of major metabolites of fatty acids, sugars, and amino acids of the Krebs cycle are affected by BID following HFD feeding (Figures 6 and 8). Although all of these metabolites could contribute to the metabolic phenotype of *bid* ^*−/−*^ mice, only the levels of some intermediates of the Krebs cycle are also affected by antibiotics treatment (Figure 8B), suggesting that this metabolic pathway could be a major mechanistic point where signals from BID and gut dysbiosis affect each other.

Because the Krebs cycle occurs in the mitochondria, this organelle could be the key site for BID and gut microbial regulation. The apoptotic truncated form of BID, known as tBID, can cause mitochondrial dysfunction to trigger apoptosis.^[^ ^9^, ^10^ ^]^ However, BID may affect mitochondrial function in a nonapoptotic way. One study showed that tBID could alter mitochondrial fatty acid oxidation flux by inhibiting carnitine palmitoyltransferase‐1.^[^ ^44^ ^]^ BID is also important in maintaining normal mitochondrial cristae organization.^[^ ^45^ ^]^ BID deficiency in the myeloid progenitor cells or cardiac cells leads to decreased respiration, increased oxygen consumption, and decreased adenosine triphosphate production as measured *in vitro*.^[^ ^45^ ^]^ However, we have not detected significant expressional differences in genes related to fatty acid oxidation between WT and *bid* ^*−/−*^ livers (Figure S7). We had also failed to detect a consistent difference in mitochondrial consumption of oxygen and fatty acid oxidation between WT and *bid* ^*−/−*^ hepatocyte mitochondria (data not shown).

Thus, there might be other ways that BID could affect lipid metabolism. Interestingly, BID was previously found to possess a lipid transfer activity.^[^ ^46^ ^]^ BID could still affect metabolic functions by regulating cell death. BID can mediate apoptosis in adipocytes^[^ ^47^ ^]^ or pancreatic β cells,^[^ ^48^, ^49^ ^]^ whose demise may affect metabolic syndrome in some ways. Other BH3‐only molecules that affect metabolisms are BAD, which can be associated in a glucokinase‐containing complex, thereby regulating glycolysis,^[^ ^17^ ^]^ insulin secretion,^[^ ^18^, ^19^ ^]^ and β‐cell function,^[^ ^19^, ^21^ ^]^ and BIM, which can affect lipogenesis and lipid oxidation to affect body weight and insulin sensitivity following HFD.^[^ ^20^, ^22^ ^]^ Thus, different BH3‐only BCL‐2 family molecules may affect metabolisms through different mechanisms.

In conclusion, this study presents a promoting role of BID in diet‐induced obesity and hepatic steatosis in a way critically intercepted by GM. The interaction with GM is a significant feature of BID in regulating metabolism among the BH3‐only BCL‐2 family molecules. The present study also suggests that targeting BID can be a potential therapeutic approach for metabolic syndrome.

## FUNDING INFORMATION

Supported in part by the National Institute of Diabetes and Digestive and Kidney Diseases (R01‐DK116605) and the Louisiana Clinical and Translational Science Center fund (U54 GM104940).

## CONFLICT OF INTEREST

Nothing to report.

## Supporting information


**Appendix S1** Supporting Information
