# Supplementary material for: Promotion of diet‐induced obesity and metabolic syndromes by BID is associated with gut microbiota
Source: Hepatol Commun. 2022 Nov 15;6(12):3349–62. doi: 10.1002/hep4.2052 (PMC9701492; doi:10.1002/hep4.2052)
Supplement: Supplementary file 1 — Appendix S1 Supporting Information [file HEP4-6-3349-s001.docx]

**Promotion of Diet-induced Obesity and Metabolic Syndromes by BID is Associated with Gut Microbiota**

Shengmin Yan^1,2, *^, Jun Zhou^2,3, *^, Hao Zhang^2 *,#^, Zhen Lin^1^, Bilon Khambu^1, 2^, Gang Liu^1^, Michelle Ma^1^, Xiaoyun Chen^2^, Naga Chalasani^4^, and Xiao-Ming Yin^1,2, $^

^1^ Department of Pathology and Laboratory Medicine, Tulane University School of Medicine

New Orleans, LA 70112, USA

^2^Department of Pathology and Laboratory Medicine, Indiana University School of Medicine

Indianapolis, IN 46202, USA

^3.^Department of Emergency Medicine, The Second Xiangya Hospital, Central South University, Changsha, China

^4^Department of Medicine, Indiana University School of Medicine

Indianapolis, IN 46202, USA

***. These authors are co-first authors**

**$. Address correspondence to:**

Xiao-Ming Yin, MD, PhD

Department of Pathology and Laboratory Medicine

Tulane University School of Medicine,

New Orleans, LA 70112

504-988-1170 (phone)

504-988-7389 (fax)

xmyin@tulane.edu (e-mail)

#Current address:

Hao Zhang, MD, PhD

Digestive Health Institute

University of Illinois

611 W. Park. St.

Urbana, IL 61801


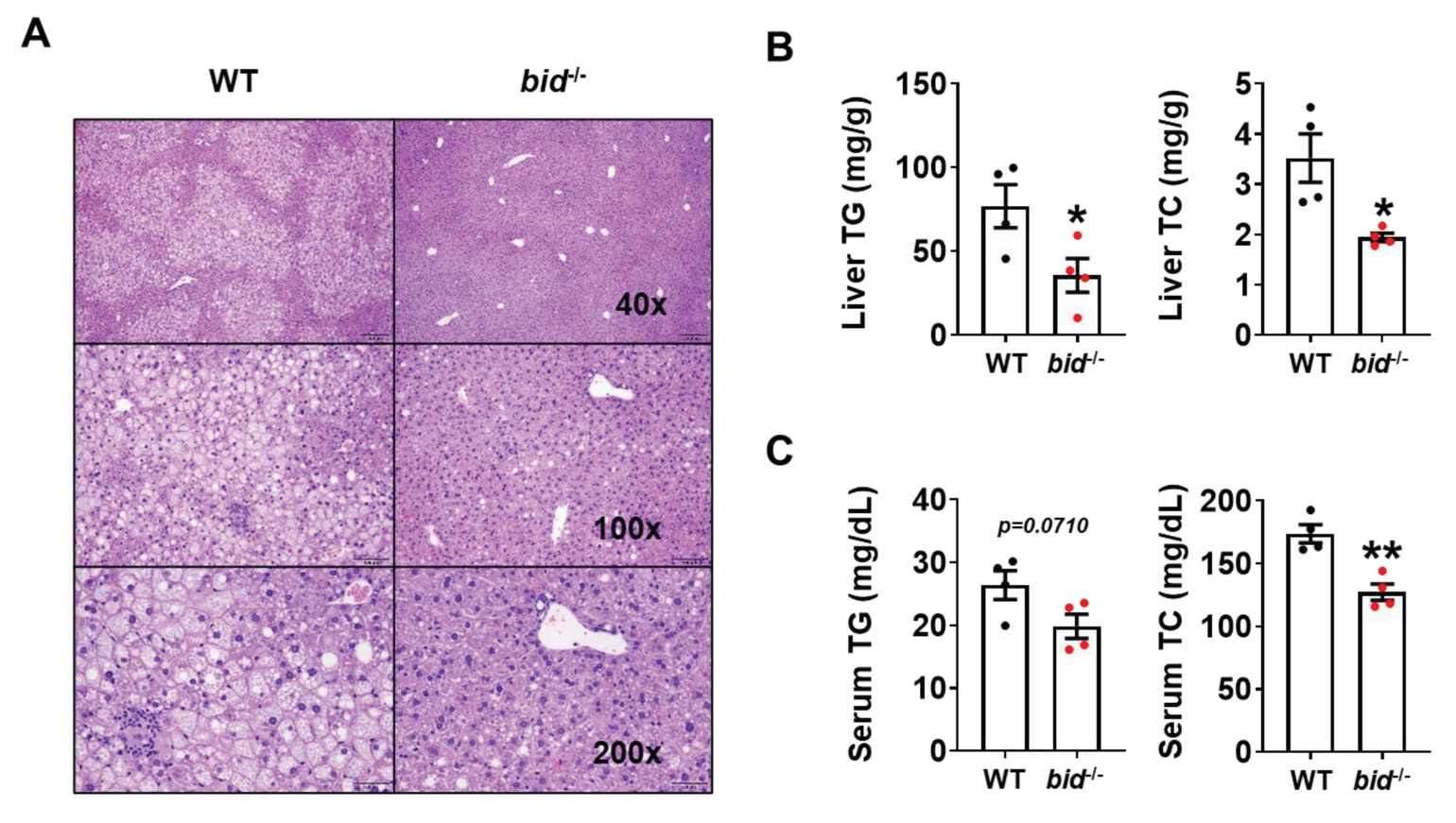


**Fig.S1. *bid*^-/-^ mice are resistant to HFD-induced obesity and hepatic steatosis.**

Male wild type (WT) and *bid*-deficient (*bid^-/-^*) mice were on high-fat diet (HFD) for 20 weeks (n=4). (A). Representative images of hepatic H&E staining (40x,100x, and 200x) showed more severe steatosis in the wild type livers. (B-C). Triglyceride (TG) and total cholesterol (TC) levels in the liver (B) and the serum (C) (n=4). Data were shown as means ± S.E, * *p*<0.05, ** *p*<0.01.


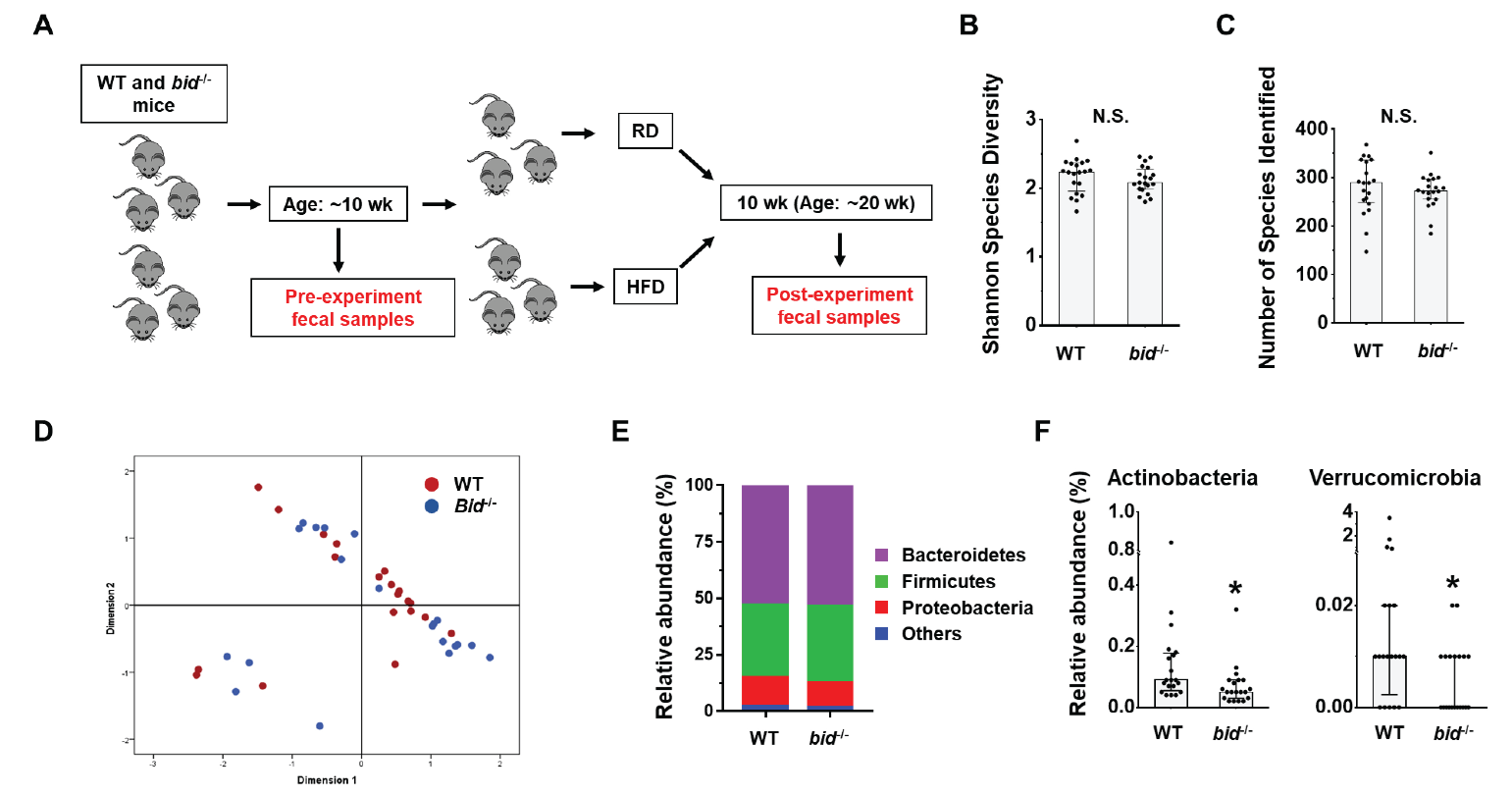


**Fig.S2. The GM composition in wild type and *bid*^-/-^ mice before HFD feeding.**

Male wild type (WT) and *bid*-deficient (*bid^-/-^*) mice were on high-fat diet (HFD) for 10 weeks. (A) Scheme of feeding and timing of fecal sample collection. (B-C) Shannon species diversity (B) and the number of identified species (C) were comparable between the WT and *bid^-/-^* mice before the administration of HFD. (D). Principal coordinates analysis (PCoA) based on relative abundance at species level shows a comparable profile of GM between WT and *bid^-/-^* mice. (E). Proportion of dominant bacteria at the phylum level. (F). Proportion of Actinobacteria and Verrucomicrobia was decreased in *bid^-/-^* mice. Data were shown as median with interquartile range, n=20. Statistical significance was determined by Kruskal-Wallis Test following Mann-Whitney Test, **p*<0.05.


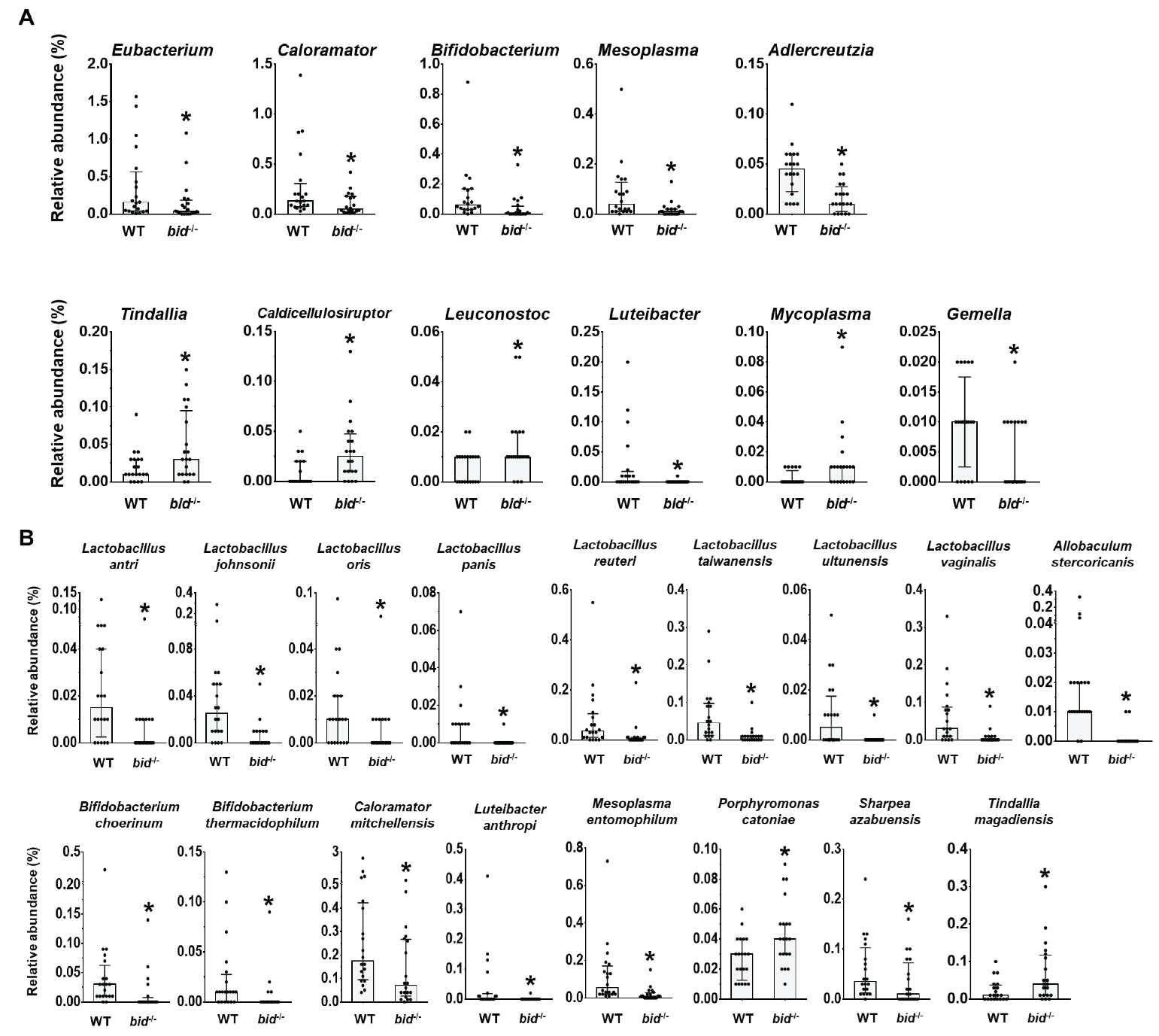


**Fig.S3. Disproportionated low abundance gut bacteria in RD-fed *bid^-/-^* mice at the genus level.**

Disproportionated low abundance bacteria in regular diet (RD)-fed male mice at the genus level (A), and at the species level (B). Data were shown as median with interquartile range, n=20/group. Statistical significance was determined by Mann-Whitney Test, **p*<0.05.


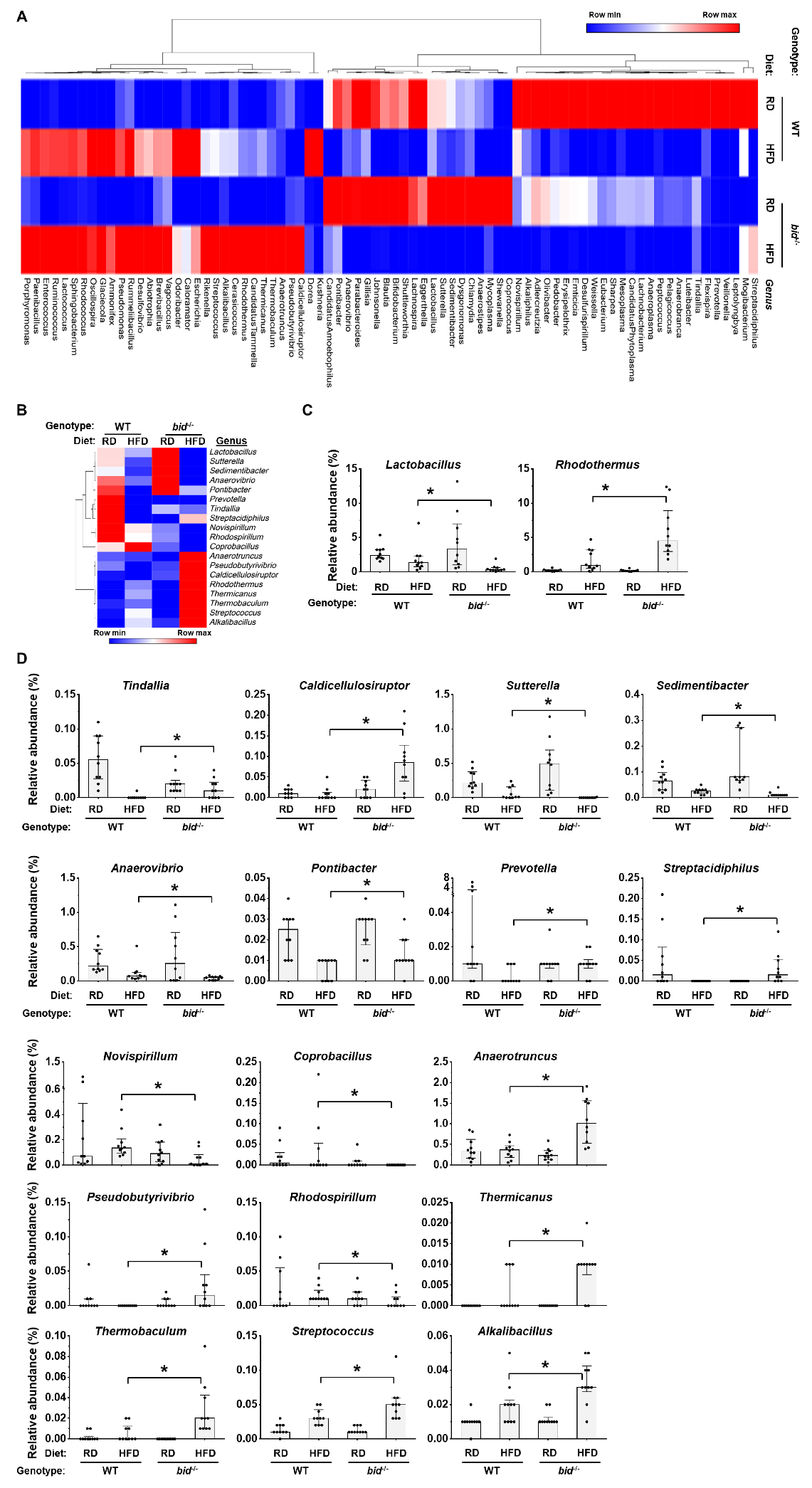


**Fig.S4. Disproportionated gut bacteria in mice following HFD treatment at the genus level.**

Male wild type (WT) and *bid*-deficient (*bid^-/-^*) mice were on high-fat diet (HFD) for 10 weeks. (A). Heatmap of disproportionated bacteria following HFD feeding. Seventy-eight bacteria with significantly changed relative abundance at the genus level were shown. (B). Bacteria with different enrichment in HFD-fed WT and *bid^-/-^* mice as selected from panel A. (C-D). Relative abundance of high abundance bacteria (C), and of low abundance bacteria (D) that showed different enrichment in HFD-fed WT and *bid^-/-^* mice as displayed in panel B. Data were shown as median with interquartile range, n=10/group. Statistical significance was determined by Kruskal-Wallis Test following Mann-Whitney Test, **p*<0.05.


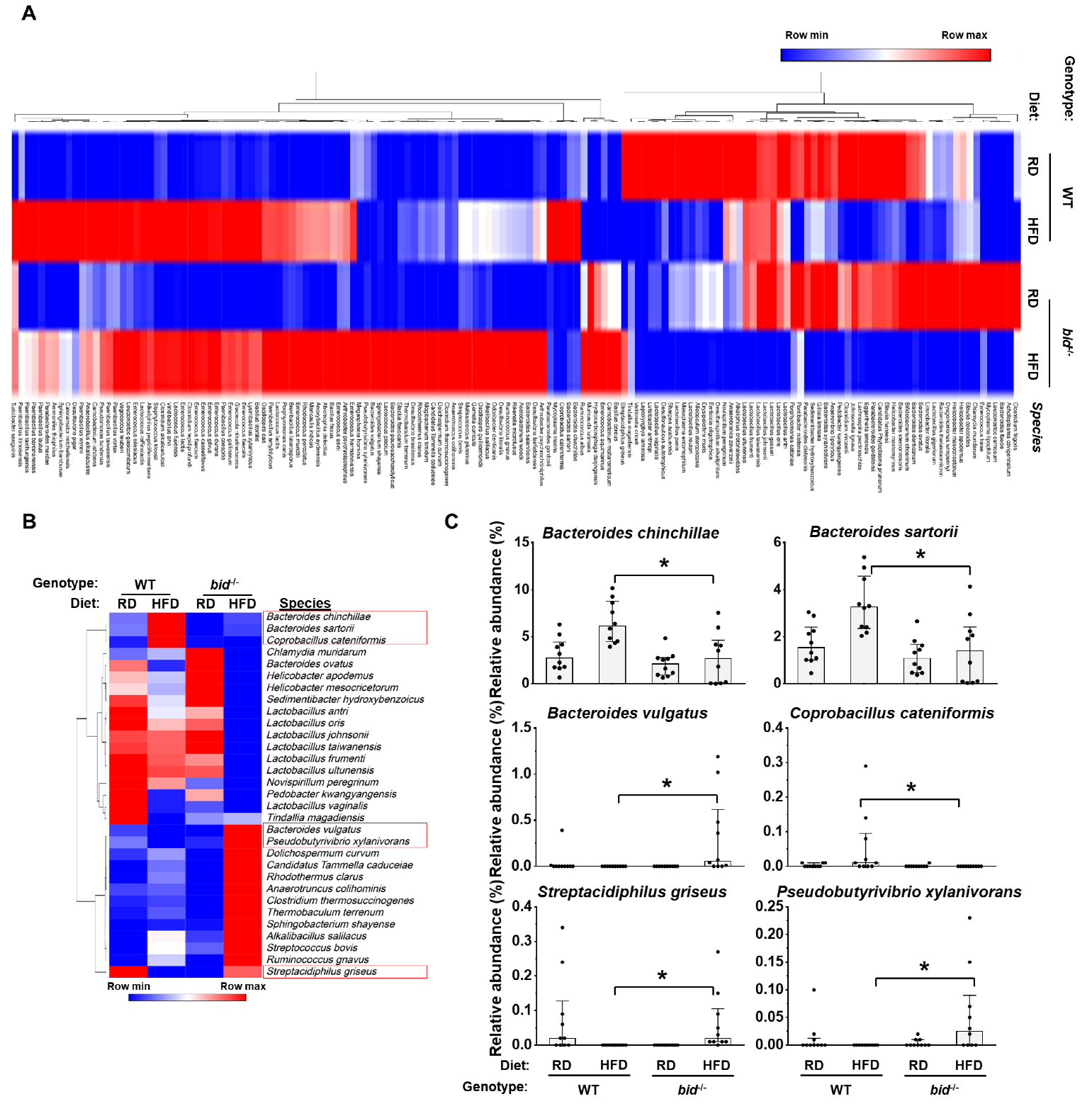


**Fig.S5. Disproportionated gut bacteria in mice following HFD feeding at the species level.**

Male wild type (WT) and *bid*-deficient (*bid^-/-^*) mice were on high-fat diet (HFD) for 10 weeks. (A). Heatmap of disproportionated bacteria following HFD feeding. One hundred forty-nine bacteria with significantly changed relative abundance at the species level were shown. (B) Bacteria with different enrichment in HFD-fed WT versus *bid^-/-^* mice as selected from panel A. (C) Relative abundance of representative bacteria from panel B that showed different enrichment in HFD-fed WT and *bid^-/-^* mice. Data were shown as median with interquartile range, n=10/group. Statistical significance was determined by Kruskal-Wallis Test following Mann-Whitney Test, **p*<0.05.


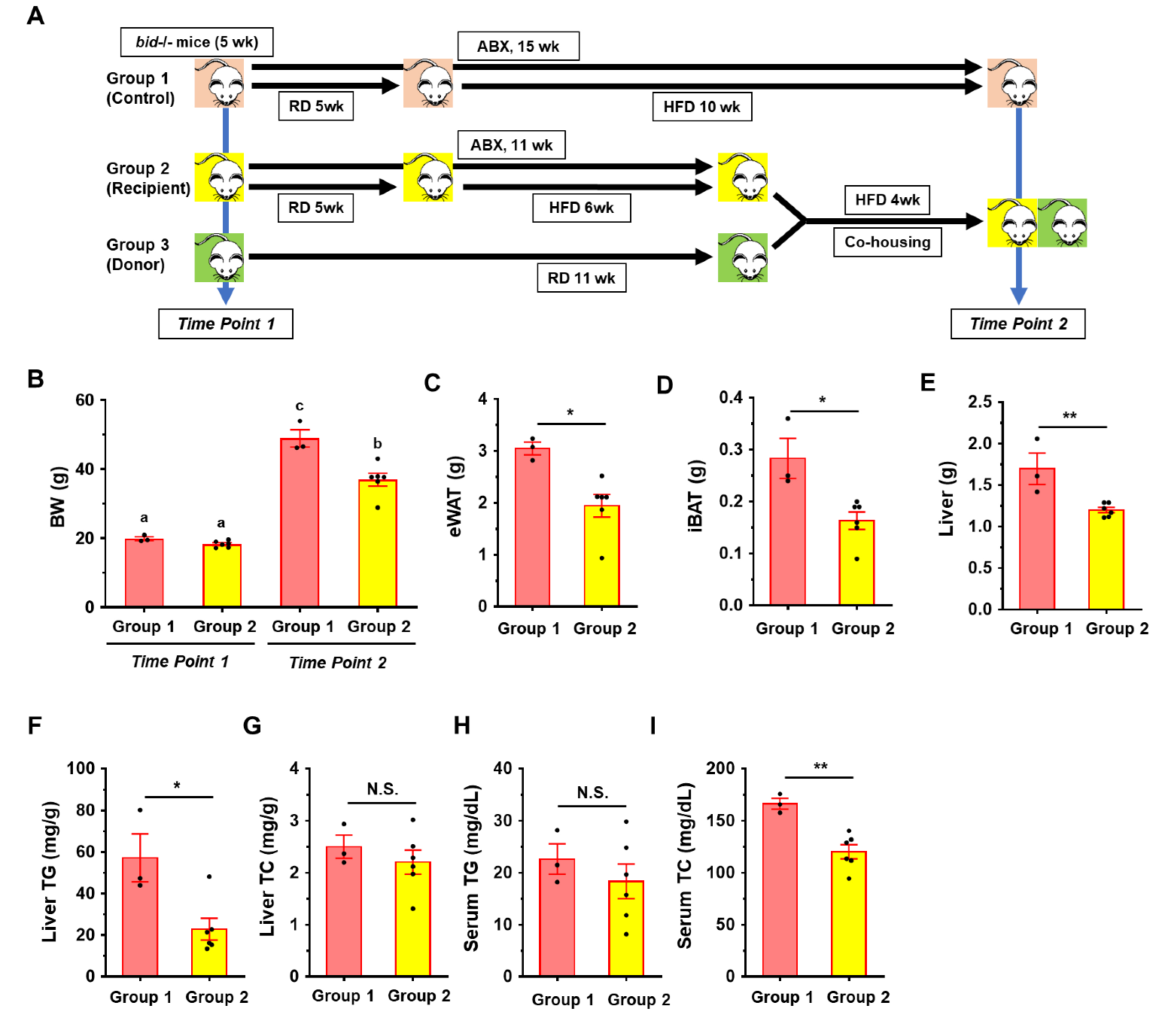


**Fig. S6. Co-housing with naive mice reversed obese phenotype in ABX-treated mice.**

(A). Scheme of HFD feeding, ABX treatment and co-housing. Control and recipient *bid*-deficient (*bid^-/-^*) mice (Group-1 and Group-2) had been exposed to ABX (0.5g/L Neomycin Sulfate and 1g/L Ampicillin, Sodium Salt), and HFD. Donor *bid^-/-^* mice (Group-3) had not been exposed to ABX before co-housing. Every three Group-2 mice were co-housed with two Group-3 mice. All analyses were performed for Group-1 and Group-2 mice at the end of experiment (Time Point 2) except for the body weight, which was also measured at Time Point 1. (B-E) Weight of the body (B), eWAT (C), iBAT (D) and liver (E) were determined at two time points for body weight, and at time point 2 for eWAT, iBAT and the liver. (F-I) Hepatic and serum triglycerides, and hepatic and serum total cholesterol were measured at Time Point 2. n=3-6/group. Statistical significance was determined by One-way ANOVA followed by Duncan’s post-hoc test (B) or by Student’s t-test (C-I). Groups with different letters had significant differences (*p<0.05*). **: *p*<0.01.


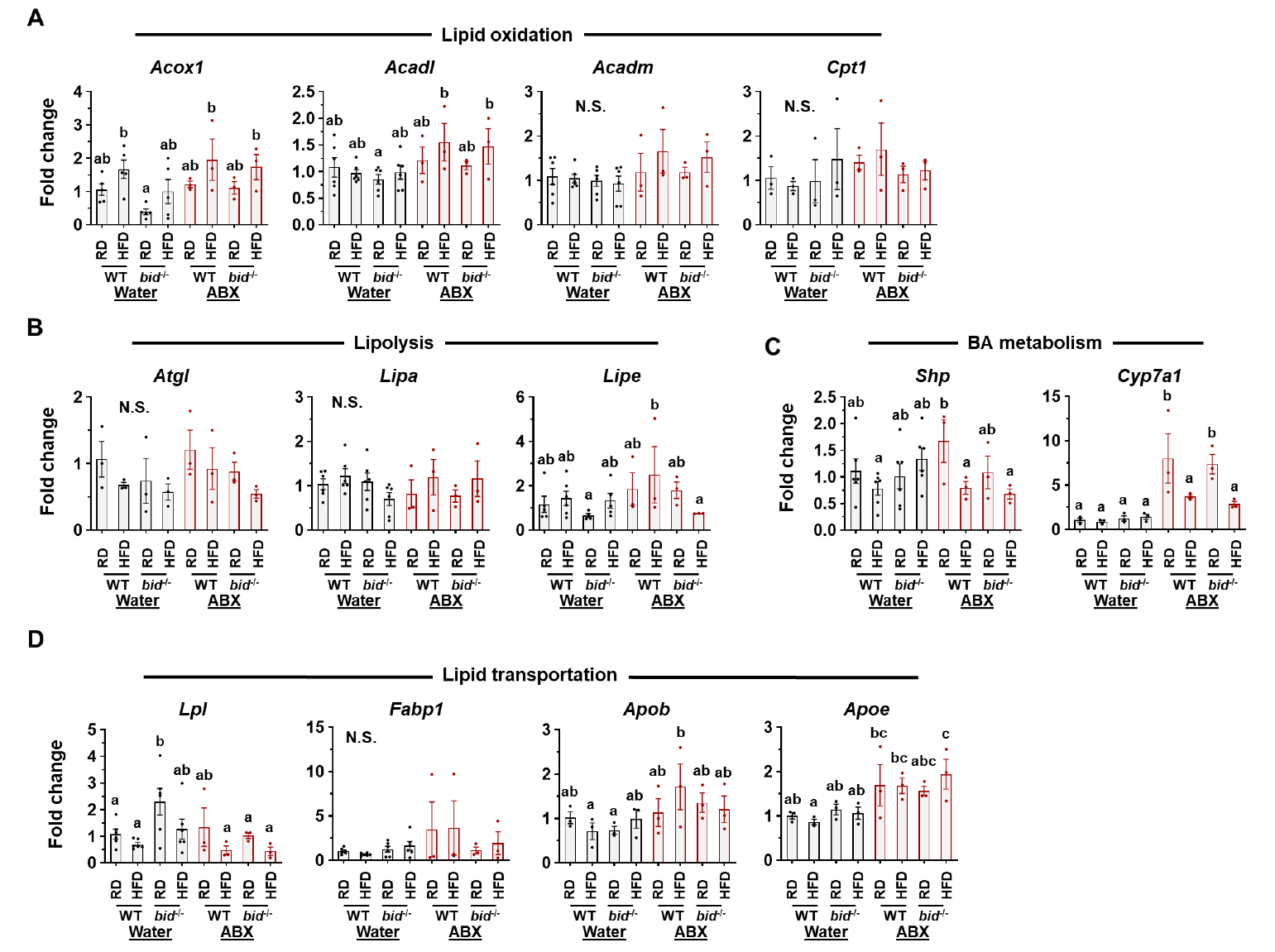


**Fig. S7. Hepatic expression of genes related to lipid metabolism.**

Male wild type (WT) and *bid*-deficient (*bid^-/-^*) mice were on regular chow diet (RD) or high-fat diet (HFD) for 10 weeks with or without antibiotics (ABX) treatment (Fig. 4A). Hepatic expression of genes related to lipid oxidation (A), lipid lipolysis (B), bile acid (BA) metabolism (C), or lipid transportation (D), were assessed in mice without (n= 5-6) or with ABX treatment (n=3). Data were shown as means ± S.E. Statistical analysis was performed by One-way ANOVA followed by Duncan’s post-hoc test. Groups with different letters had significant differences (*p<0.05*). N.S.: not significant. *Acadl*: acyl-CoA dehydrogenase long chain; *Acadm*: acyl-CoA dehydrogenase medium chain; *Acox1*: acyl-CoA oxidase 1; *Apob*: apolipoprotein B-100; *Apoe*: apolipoprotein E; *Atgl*: adipose triglyceride lipase; *Cpt1*: carnitine palmitoyltransferase-1; *Cyp7a1*: cytochrome P450 family 7 subfamily A member 1; *Fabp1*: fatty acid binding protein 1; *Lipa*: lipase A, lysosomal acid type; *Lipe*: hormone-sensitive lipase; *Lpl*: lipoprotein lipase; *Shp*: small heterodimer partner.


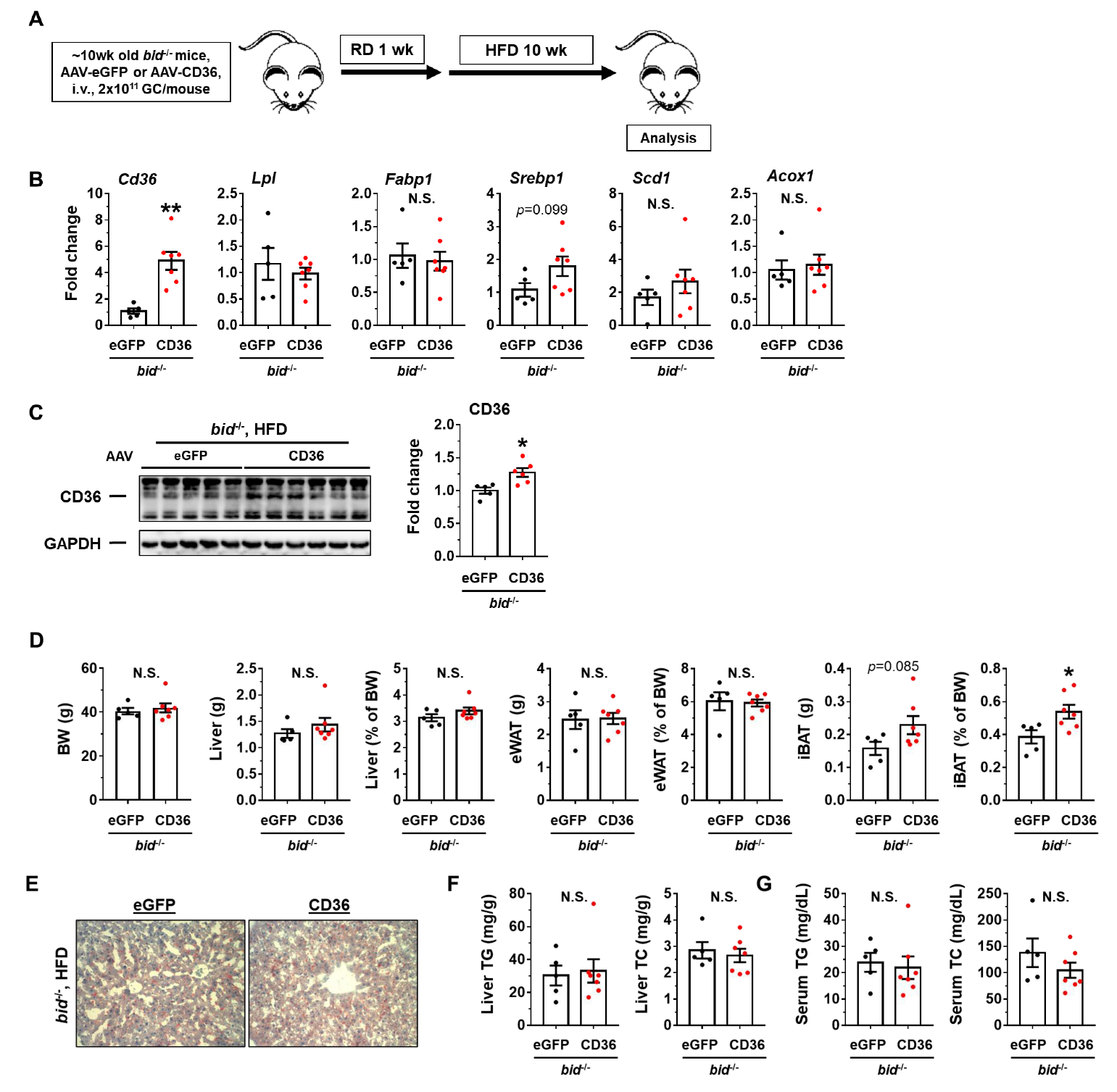


**Fig.S8. Overexpression of CD36 in *bid^-/-^* liver did not promote HFD-induced steatosis.**

(A). Scheme of AAV injection and HFD feeding. 10-week-old male *bid*-deficient (*bid^-/-^*) mice were randomly divided into 2 groups and intravenously given AAV-cluster of differentiation 36 (CD36) or AAV-enhanced green fluorescent protein (eGFP) respectively. Mice were then given continually regular chow diet (RD) feeding for 1 week, followed by high-fat diet (HFD) feeding for 10 weeks. Mice were sacrificed and samples were collected after 10 weeks. (B). Hepatic expression of cluster of differentiation 36 and other genes related to lipid metabolism (n=5-7). (C). Protein level of CD36 in the liver following injection of AAV constructs was analyzed by immunoblot assay and quantified by densitometry, (n=5-6). (D). The weight of the whole body (BW), the liver, the epididymal white adipose tissue (eWAT) and the interscapular brown adipose tissue (iBAT) in eGFP- or CD36-overexpressing *bid^-/-^* mice following HFD feeding (n=5-7). (E). Representative images of hepatic Oil Red O staining (200x). (F-G). Triglyceride (TG) and total cholesterol (TC) levels in livers (F) and serum (G)(n=5-7). Data were shown as means ± S.E. **p*<0.05, ***p*<0.01, Student *t*-test.
